# Does a tailored intervention to promote adherence in patients with chronic lung disease affect exacerbations? A randomized controlled trial

**DOI:** 10.1186/s12931-019-1219-3

**Published:** 2019-12-03

**Authors:** Claudia Gregoriano, Thomas Dieterle, Anna-Lisa Breitenstein, Selina Dürr, Amanda Baum, Stéphanie Giezendanner, Sabrina Maier, Anne Leuppi-Taegtmeyer, Isabelle Arnet, Kurt E. Hersberger, Jörg D. Leuppi

**Affiliations:** 1 University Clinic of Medicine, Cantonal Hospital Baselland, Rheinstrasse 26, CH - 4410 Liestal, Switzerland; 2 Department of Pharmaceutical Sciences, University of Basel, Basel, Switzerland; 3 Faculty of Medicine, University of Basel, Basel, Switzerland; 4 Centre for Primary Health Care, University of Basel, Basel, Switzerland; 5 Department of Clinical Pharmacology and Toxicology, University Hospital Basel, Basel, Switzerland

**Keywords:** Chronic disease management, Compliance, Randomised controlled trial, Reminders

## Abstract

**Background:**

Poor medication-adherence is common in chronic lung patients, resulting in reduced health-outcomes and increased healthcare-costs. This study aimed to investigate the impact of an acoustic reminder and support calls on adherence to inhaled therapy in asthma and COPD patients and to determine their effect on exacerbations.

**Methods:**

This single-blinded randomized controlled trial investigated asthma and COPD patients during 6 months in an ambulatory setting. The intervention consisted of daily alarm clock and support phone calls, whenever use of rescue medication doubled or inhaled medication was not taken as prescribed. Primary outcome was time to next exacerbation. Frequency of exacerbations, adherence to inhaled medication and quality of life scores were secondary outcomes. Cox and Poisson regression were used to determine intervention effect on time to exacerbation and frequency of exacerbations, respectively.

**Results:**

Seventy-five participants were assigned to the intervention group and 74 to usual follow-up care. During a median follow-up of 6.2 months, 22 and 28% in the intervention and control groups respectively, experienced at least one exacerbation. Intervention had no effect on time to first exacerbation (HR 0.65, 95% CI 0.21 to 2.07, *P* = .24), but showed a trend toward a 39% decreased frequency of exacerbations (RR = 0.61, 95% CI 0.35 to 1.03, *P* = .070) for the adjusted models, respectively. The intervention group had significantly more days with 80–100% taking adherence regarding puff inhalers (82 ± 14% vs. 60 ± 30%, *P* < .001) and dry powder capsules (90 ± .10% vs. 80 ± 21%, *P* = .01). Timing adherence in participants using puff inhalers was higher in the intervention group (69 ± 25% vs. 51 ± 33%, *P* < .001). No significant differences in QoL were found between the two groups.

**Conclusion:**

Participants assigned to the intervention group had significantly better taking and timing adherence of inhaled medication resulting in a trend towards a decreased frequency of exacerbations. However, no effect on time to next exacerbation was observed.

**Trial registration:**

ClinicalTrials.gov: NCT02386722, Registered 14 February 2014.

## Background

Asthma and chronic obstructive pulmonary disease (COPD) represent a major economic burden [1]. Poor adherence to prescribed medication is common in patients with asthma and COPD, varying from < 23–70% [2–5] . According to the World Health Organisation (WHO), adherence is defined as “the extent to which a person’s behaviour corresponds with the agreed recommendations from a healthcare provider” [6]. Suboptimal or non-adherence to inhaled therapies has been shown to be associated with increased rates of morbidity, healthcare expenditures, hospitalisations, and mortality [7, 8]. Moreover, quality of life (QoL) is reduced [9] and medical care is used more often due to deterioration of symptoms and recurrent exacerbations [10].

Approximately 50–75% of healthcare expenditures related to COPD are caused by exacerbations [11], which often require hospital stays, physician visits, and additional medication. Moreover, exacerbations adversely affect patients’ quality of life, lung function, and mortality [12]. A recent Swiss study has shown that a comprehensive self-management asthma education programme can improve asthma control and patients’ outcomes [13, 14]. Besides, it is noteworthy that higher adherence rates have been associated with lower exacerbation rates in patients with asthma [15, 16] and COPD [17].

Thus, sufficient adherence to medication is a prerequisite for the achievement of therapeutic success in chronic diseases. Various interventions and strategies for improving adherence have been described. Interventions which aimed at improving adherence are most successful when the use of electronic devices and feedback on patients’ adherence behaviour are combined [18]. However, an intervention should be tailored to the individual patient’s needs [19].

Therefore, the aims of this study were to investigate the effect of a patient-tailored intervention on adherence to inhaled therapy in patients with asthma and COPD and to determine the resulting effect on time to next exacerbation as well as exacerbation rates in the investigated study population.

## Methods

### Study design

This single-blinded randomized controlled study was conducted in an ambulatory setting in Switzerland (ClinicalTrials.gov: NCT02386722). The study details and baseline characteristics of the participating patients have been published previously [20, 21]. In brief, 169 adult participants with an established asthma-diagnosis according to the Global Initiative for Asthma (GINA) guidelines [22] and/ or an established COPD diagnosis according to the Global Initiative for Chronic Obstructive Lung Disease (GOLD) guidelines (severity GOLD I-IV based on the international GOLD-Criteria [23] were included in the study. Patients were recruited from several hospitals in the Basel region and from private practicing pulmonologists. They were followed-up every 2 months for a total of 6 months. All participants had to have experienced at least one exacerbation within the previous year and had to be treated with daily inhaled medication (controller medication for daily maintenance treatment). Participants remained on the treatment plan initiated by their general practitioner (GP). Written informed consent was obtained from every participant. Depending on the prescribed medication, all participants were equipped with Smartinhaler devices for puff inhalers such as metered dose inhalers and multidose dry powder inhalers (Adherium Ltd., Auckback, New Zealand) and/or with Electronic Monitoring System (POEMS) consisting of a printed, self-adhesive polymer film affixed to a multidose punch card (Pharmis GmbH, Beinwil am See, Switzerland) that had been prefilled with dry powder capsules. Each inhalation device actuation of the Smartinhaler was saved with date and time and data were transferred daily to an online database via wireless internet connection. Every time the patient broke a loop for taking the capsules, date and time were recorded on a microchip, which was read out every 2 weeks when participants brought back the empty punch card. In order to detect false device application, all participants were asked to demonstrate their inhalation technique with all prescribed devices to the investigator by using placebo devices (to avoid overdosing) at every study visit. The study was carried out in accordance with the Declaration of Helsinki and Good Clinical Practice guidelines. The local ethics committee northwest/central Switzerland (registry number: EK-269/13) approved the study.

### Randomisation

A randomization list with study group allocation was generated by using R (RStudio, Boston, US). Participants were randomly assigned in a block size of two to the intervention or the control group. This reduced the risk of a season effect between the two study groups. Furthermore, participants were not aware of which group they had been randomized to (single-blinded). Patients were neither informed that there were two study groups nor about being allocated to a study group with or without supervision and support.

### Study intervention

The intervention consisted of an audio-reminder generated by an app (for Smartinhaler devices) or an alarm clock (for POEMS) that were directly transferred to the participants’ smartphones. Participants were allowed to choose the inhalation times themselves, depending on their GP’s treatment plan, their personal habits and daily routine. Since in most cases the inhalation times during the morning or the evening do not differ even with several inhalation devices, patients received only one reminder which was valid for all devices prescribed at that time. In cases where patients were used to inhaling their medication at several time points during the day, a reminder was set for each inhalation. The audio-reminder and alarm clock generated for the study by the smartphone had to be switched off by the participants, which confirmed that the signal had been received. Participants in the intervention group received support calls from the study pharmacist or study nurses when the use of rescue medication doubled or when the medication was not inhaled as prescribed for more than two consecutive days (only for puff inhalers). During the support calls, the patients were first made aware of the missing inhalation during the past days and then, depending on the answer and the resulting reason for the non-adherence, the problems were specifically addressed in a tailored fashion with the aim to ensure regularity of inhalation. The content of the support calls was not based on a pre-prepared template. However, in order to standardize the process of the calls, the answers to the individual non-adherence problems were discussed in advance between the pharmacist and the study nurse who carried out the support calls. The content and duration of the calls were documented after each intervention. All participants (puff inhalers and dry powder capsules) also received a feedback on their intake pattern at each clinical visit, in the form of a visualization graph (Additional file 1).

Participants assigned to the control group did neither receive any reminder nor additional assistance or feedback regarding their medication adherence behaviour. Adherence data of these participants were analysed at the end of the study period and were not examined by the investigators during the study.

### Outcome assessment

Sociodemographic variables such as age, gender and civil status were obtained by a generic questionnaire at the baseline visit. Smoking status, pack years (py) and body mass index (BMI) were assessed together with disease-related aspects such as allergies, number of exacerbations and hospitalisation during the previous 12 months.

The primary outcome was “time to next asthma or COPD exacerbation”. Exacerbation was defined as acute-onset worsening of the patient’s condition beyond day-to-day variations requiring interaction with a healthcare provider [24]. Time to next exacerbation was defined as the number of days between study begin and the first exacerbation. For patients treated at the Cantonal Hospital Baselland, internal medical records could be screened to collect the needed information regarding exacerbations. For patients treated elsewhere, the treating physician was contacted to collect the necessary information. The following secondary outcomes were recorded and analysed: Frequency of exacerbation (defined as the number of exacerbations during the study period), the number of severe exacerbations which led to a hospitalisation, timing- and taking-adherence and health-related quality of life (QoL).

Adherence was quantified by using Smartinhalers and POEMS devices [20], starting at the baseline visit and continuing until the end of the study. Smartinhalers were used for the inhalation with puff inhalers (metered dose inhalers, Turbohaler, Discus and Ellipta®). Once the devices were installed on the inhalers, participants were able to use their medication as usual. POEMS were used for inhalation with dry powder capsules. When any therapy adjustments with additional inhaled medication or with a new treatment plan from the treating physician occurred, patients were instructed to inform the study team immediately. The study team prepared the necessary adjustments and informed the patients about the next steps (for example new Smartinhaler-delivery medication change to be carried out by the patients themselves) in order to be able to guarantee a seamless measurement of the adherence.

Objective adherence was quantified based on the following pre-specified criteria [25]:

- Taking adherence = [number of puffs inhaled during 24 h / number of puffs prescribed during 24 h) × 100. Correct taking adherence was considered when taking adherence was between 80 and 100% (target range), based on previous studies [26].
- Timing adherence = [number of correct dosing intervals during 24 h / number of dosing intervals during 24 h) × 100; correct dosing intervals was defined as an interval within a grace period of 25%, i.e. between
  - ◦ 18–30 h for once daily dosing,
  - ◦ 9–15 h for twice daily dosing and
  - ◦ 6–10 h for three times daily dosing.
- Gaps = [number of days without inhalation during the study period / number of days of the study period] × 100.
- Maximal gap length = longest period of time (in days) without inhalation.

For the adherence calculation only the regularly inhaled medication was considered. Rescue inhalers which were only inhaled when needed, were not included in the adherence calculations.

For the case that patients had multiple devices, adherence was calculated for each single device and then the median was calculated for every single day. Adherence of puff inhalers and dry powder capsules were calculated and evaluated separately, due to the fact the adherence was assessed with different methods.

Health-related QoL was assessed using the St. George Respiratory Questionnaire (SGRQ) [27]. Permission for the use of the SGRQ has been obtained by the St George’s University of London (St George’s Hospital Medical School).

Missing values of tests or clinical examinations were not included in the corresponding results, but were no reason to exclude the patients from the study.

### Sample size calculation

Power calculation was based on the primary outcome measure “time to next exacerbation.” Details of the sample size calculation can be found in the published protocol [20]. Briefly, an 80% power to detect a hazard ratio (HR) of 0.36 for time to next exacerbation in the intervention group was expected by including 70 patients in each group.

### Statistical analysis

Statistical analyses were performed using the software R 3.1.3 [28] and the SPSS software package (version 23, IBM, Germany). Statistical significance was set at the 5% level. Data are presented as mean and standard deviation (SD) or number and percentage (%). Differences between intervention and control group were assessed using t-test for continuous parametric variables, and the Mann-Whitney U-test for non-parametric variables. For categorical variables, the Pearson’s chi-square test was used. Poisson regression was used to analyse the number of exacerbations within the study period (uncensored across the entire 6-months) with respect to the effect of being in the intervention group versus the control group. Time to next exacerbation was assessed using survival analyses. Median follow-up was calculated across censoring time (i.e. participants without exacerbation). Univariate analyses were performed based on the Cox proportional hazards model (censored at the first exacerbation), using group as the independent variable. Results are reported as HR with a corresponding confidence interval (CI) of 95% and *p*-value. Exacerbation-free survival curves for the two groups were estimated and visualized by the Kaplan-Meier product limit method and compared using the log rank test.

Considering that the intervention and control groups differed in some characteristics at baseline, the association of these variables with both the incident exacerbations and time to next exacerbation were assessed using univariate Poisson regression and Cox regression, respectively (Additional file 2). Variables with a significant association (*P* < .05) in univariate analysis were then entered into the multivariate model as possible confounders of the association between allocated group and incident exacerbations or time to next exacerbation.

The robust nonparametric analysis of longitudinal adherence data was conducted with the nparLD r package (function f1.ld.f1) to determine the effects of the factors time (1–200 days) and group (control and intervention) on different measures of adherence in percent (taking, timing) [29]. Such nonparametric methods are also robust with respect to outliers, missing data and small sample sizes.

## Results

**Fig. 1:**
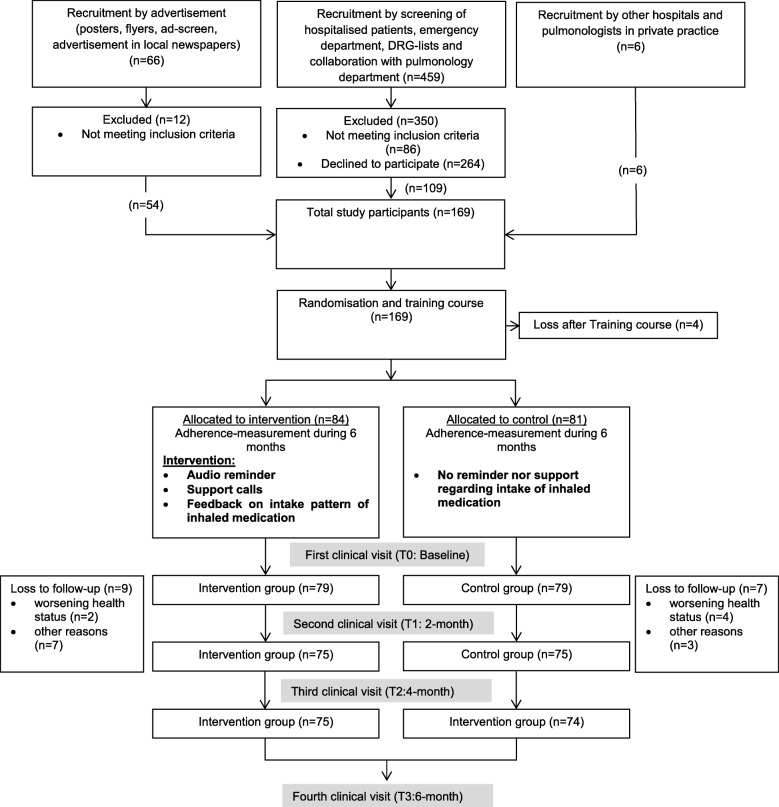
Flow chart of the study

Figure 1 provides an overview of the study flow. One hundred sixty-nine participants met the inclusion criteria and were willing to participate in the study. Four participants withdrew consent prior to randomization so that 165 patients were assigned either to the intervention or to the control group. Further 16 participants withdrew during the follow-up due to different reasons, so that 149 participants completed the study and were investigated for the planned analysis. Three (2%) subjects had more than 25% of missing outcome data due to technical problems. Due to this small proportion of subjects with missing outcome variables and its random distribution, it was decided that there was no need to impute missing data (as originally planned in the study protocol) and to exclude these subjects from the Poisson regressions and robust nonparametric analysis of longitudinal data. Instead, we conducted a complete case analysis. Cox regression was performed with all subjects (*n* = 149).

### Baseline characteristics

Baseline patient characteristics together with the prescribed medication at baseline are summarized in Table 1. Participants in the intervention group were younger, smoked less, and more of them had asthma compared to participants in the control group. Furthermore, patients assigned to the intervention group had less LAMA prescriptions.

**Table 1:** Baseline patient characteristics (*n* = 149). Values are numbers (percentages) unless stated otherwise

Characteristics	Intervention (*n* = 75)	Control (*n* = 74)
Mean (SD) age [years]	64.7 (12.4)	69.0 (8.8)
Men	46 (61)	51 (69)
Civil status		
Unmarried	7 (9)	10 (14)
Married	46 (61)	48 (65)
Divorced/widowed	22 (29)	16 (22)
Highest level of education at school [n]		
Primary school	10 (13)	11 (15)
Apprenticeship	38 (50)	52 (70)
Higher professional education	14 (19)	6 (8)
University-entrance Diploma/Commercial college	2 (3)	1 (1)
University / Collage of higher education	11 (15)	4 (6)
Employment status [n]		
Active worker	23 (31)	15 (20)
Pensioner	47 (63)	57 (77)
Never active worker	5 (6)	2 (3)
Diagnosed lung disease		
Asthma	30 (40)	16 (22)
COPD	32 (43)	45 (61)
Asthma-COPD- overlap	13 (17)	13 (17)
Smoking status		
Current smoker	16 (21.3)	12 (16.2)
Non-smokers	19 (25.3)	14 (18.9)
Ex-smokers	40 (53.3)	48 (64.9)
Mean (SD) pack-years [n]	28.6 (32.8)	41.2 (34.3)
History of allergy	35 (47)	29 (39)
Mean (SD) body mass index [kg/m^2^]	26.5 (4.2)	28.0 (5.6)
GOLD stage		
1 (FEV_1_ > 80% predicted), mild	2 (4)^a^	6 (10)^b^
2 (FEV_1_ 50–80% predicted), moderate	20 (45)^a^	24 (42)^b^
3 (FEV_1_ 30–50% predicted), severe	19 (42)^a^	21 (36)^b^
4 (FEV_1_ < 30% predicted), very severe	4 (9)^a^	7 (12)^b^
Mean (SD) FEV_1_ predicted [%]	63.9 (25.0)	56.5 (23.5)^c^
Mean (SD) FEV_1_/FVC predicted [%]	70.3 (20.7)	67.1 (22.1)^c^
Mean SGRQ symptoms score (SD)	45.7 (21.5)	48.7 (25.6)
Mean SGRQ activity score (SD)	45.2 (19.3)	52.4 (23.7)
Mean SGRQ impact score (SD)	21.8 (14.6)	29.3 (20.7)
Mean SGRQ total score (SD)	32.5 (14.7)	39.6 (20.3)
Mean (SD) inhaled medication [n]	1.9 (0.8)	2.0 (0.8)
Mean (SD) co-morbidities [n]	1.8 (1.6)	2.2 (1.7)
Co-existing illnesses [n]		
Diseases of the cardiovascular system	44 (59)	46 (62)
Endocrine, nutritional and metabolicdiseases	18 (24)	19 (26)
Diseases of the gastrointestinal system	10 (13)	8 (11)
Diseases of the musculoskeletal system and connective tissue	16 (21)	16 (22)
Mean (SD) exacerbations (last 12 months) [n]	1.7 (0.9)	2.07 (1.4)
Mean (SD) exacerbations with hospitalisation (last 12 months) [n]	0.4 (0.6)	0.7 (1.0)
Medication [n]		
LABA/LAMA combinations	10 (13)	9 (12)
LABA/ICS combinations	52 (69)	53 (72)
LAMA	26 (35)	41 (55)
LABA	14 (19)	6 (8)
ICS	10 (13)	5 (7)
SABA	32 (43)	34 (46)
SABA/SAMA combinations	2 (2.7)	2 (2.7)
Number of inhaled medication at baseline		
1	22 (29.3)	23 (31.1)
2	36 (48.0)	27 (36.5)
3	16 (21.3)	23 (31.1)
4	1 (1.3)	1 (1.4)

^a^*n* = 45; ^b^*n* = 58; ^c^*n* = 72; FEV_1_, Forced expiratory volume in 1 sec; *FVC* Forced vital capacity, *LABA* Long acting beta_2_-agonist, *LAMA* Long acting muscarinic antagonist, *ICS* Inhaled corticosteroid, *SABA* Short acting beta_2_-agonist, *SGRQ* St. George Respiratory Questionnaire

### Time to next exacerbation

Median follow-up time was 6.2 ± 0.52 months. During the study period, 37 (24.8%) participants experienced one or more exacerbations (endpoints); Sixteen (21.3%) of these were in the intervention and 21 (28.3%) were in the control group (*P* = .30, Chi square test). A longer average time to the next exacerbation was observed in the intervention compared to the control group (102 days [95% CI, 76 to 128] vs. 86 days [95% CI, 66 to 106], *P* = .19), but failed to reach a statistical significance.

**Fig. 2:**
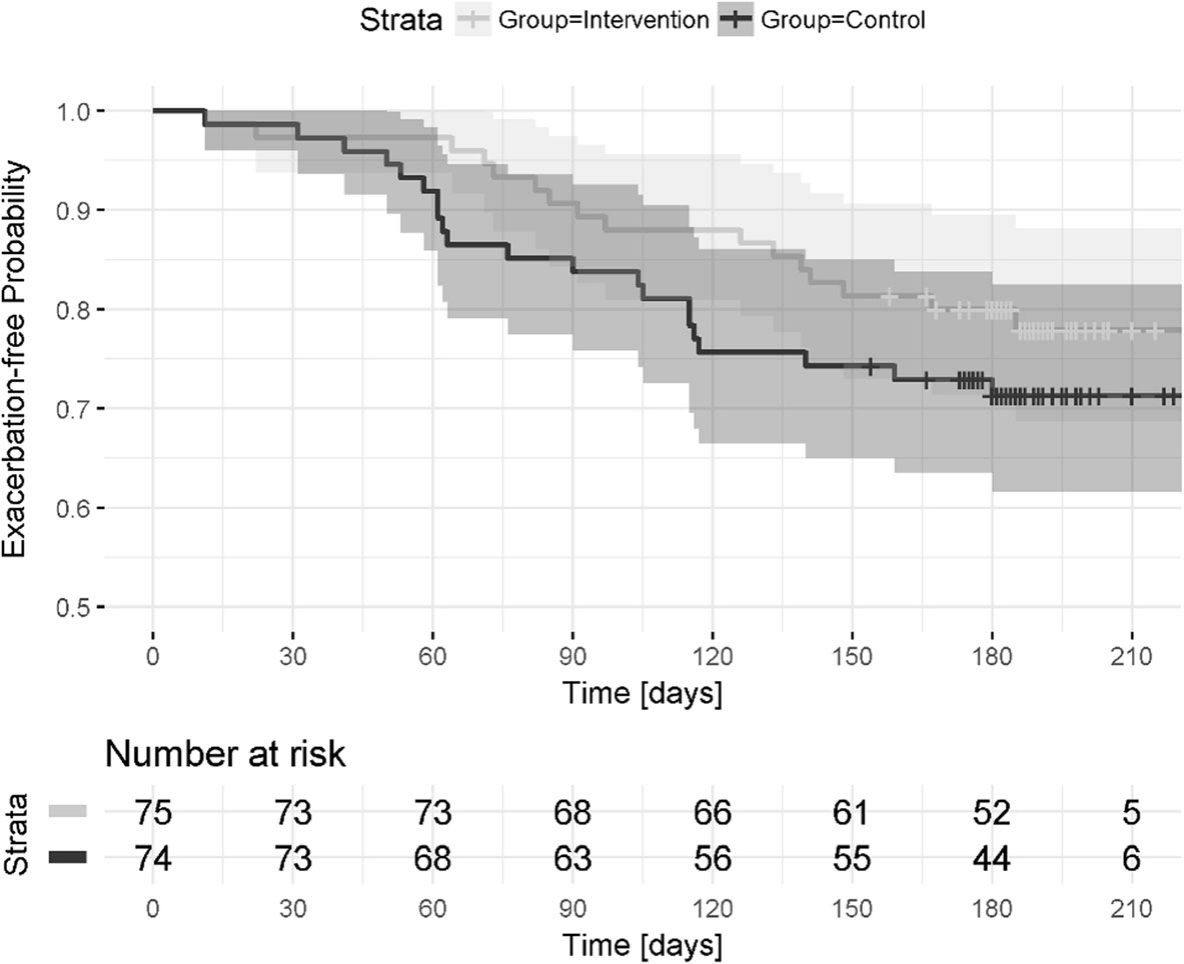
Kaplan-Meier analysis for the comparison of the time to next exacerbation in patients in the intervention compared to the control group

Survival analysis indicated that the probability of no exacerbation was 78% [95% CI: 69 to 88%] for the intervention group and 71% [95% CI: 62 to 83%] for the control group after 200 days (Fig. 2). Patients in the intervention group had a hazard ratio of 0.67 for the unadjusted model (95% CI, 0.36 to 1.33, one-sided, *P* = .14), meaning they were 0.67 times as likely as participants in the control group to experience at least one exacerbation during the study period. The multivariable analysis adjusted for age, lung disease and pack years showed a hazard ratio of 0.658 (95% CI, 0.21 to 2.07, one-sided, *P* = .0.237) (see Additional file 2 for the selection of the confounders).

### Frequency of exacerbations

In total, there were 60 exacerbations during the study period; including 12 severe exacerbations requiring hospitalisation (Table 2).

**Table 2:** Frequency of total exacerbations (*n* = 60) and severe exacerbations (*n* = 12) in the intervention and control group during the study period

Exacerbations		Number of exacerbation per person					Total number of exacerbations, n [%]
		0	1	2	3	5	
Intervention (*n* = 75)	Frequency	59	11	4	1		22 (36.7)
Control (*n* = 74)	Frequency	53	11	5	4	1	38 (63.3)
						Total:	60 (100)
Severe Exacerbations		Number of exacerbation per person					Total number of exacerbation, n [%]
		0	1	2	3		
Intervention (*n* = 75)	Frequency	70	4	1			6 (50)
Control (*n* = 74)	Frequency	70	3	0	1		6 (50)
						Total:	12 (100)

Total number of exacerbations in the intervention group: (59*0) + (11*1) + (4*2) + (1*3) =22; Total number of exacerbations in the control group: (53*0) + (11*1) + (5*2) + (4*3) + (1*5) = 38; Total number of severe exacerbations in the control group: (70*0) + (4*1) + (1*2) =6; Total number of severe exacerbations in the control group: (70*0) + (3*1) + (0*2) + (1*3) = 6

Poisson regression analysis indicated a significant effect of group for the unadjusted model. The coefficient for the intervention group was − 0.56 (95% CI, − 1.10 to − 0.04, *P* = .037). This means that the expected log count for the intervention group decreased by − 0.56 relative to the control group. In terms of relative ratios or rate ratios (RR), the results indicated an incident rate for the intervention group which was 0.57 times the incident rate for the control group (RR = 0.57, 95% CI, 0.33 to 0.95). Respectively, in terms of percent change in the incident rate of exacerbations; the results indicated a 43% decrease for the intervention group compared with the control group. The multivariable analysis adjusted for lung diseaseshowed a relative ratio of 0.61 (95% CI, 0.35 to 1.03, *P* = .070), resulting in a 39% decreased rate on the number of exacerbations (see Additional file 2 for the selection of the confounders). There was no significant effect of group for severe exacerbations (Poisson regression, *P* = .98).

### Objective adherence

Three participants had to be excluded from the adherence analysis due to more than 25% missing adherence data resulting from technical problems concerning the adherence measurement devices which failed to record the dose application. A mean of 7.17 ± 9.72 (range: 0–51) support calls per participant were performed throughout the study period in the intervention group with a mean duration of 3.0 ± 1.00 min.

#### Taking and timing adherence

Data on taking and timing adherence are provided in Table 3. Calculations for puff inhalers were based on *n* = 117 subjects and for dry powder capsules on *n* = 90 subjects. The number of monitored days was comparable for puff inhaler and dry powder capsule use in both groups.

**Table 3:** Mean percentage of days in target range for taking and timing adherence

Variable	Intervention	Control	*P* value
Taking adherence			
Mean (SD) days in target range 121for puff inhalers [%]	81.6 (14.2)^a^	60.1 (30.3)^c^	<.001
Mean (SD) days in target range 121for dry powder [%] capsules	89.6 (9.8)^b^	80.2 (21.3)^d^	.01
Timing adherence			
Mean (SD) days with correct dosing interval for puff Inhalers [%]	68.9 (25.0)^a^	50.6 (32.5)^c^	<.001
Mean (SD) days with correct dosing interval for dry powder capsules [%]	79.6 (12.9)^b^	71.7 (22.0)^d^	.052

^a^*n* = 57; ^b^*n* = 41; ^c^*n* = 60; ^d^*n* = 49

The number of days in the pre-specified adherence target range (80–100%) was significantly higher in participants assigned to the intervention compared to the control group. Timing adherence with puff inhalers was significantly higher in the intervention group (Table 3). Despite a strong trend towards greater timing adherence with dry powder capsules, the difference between the two study groups did not reach statistical significance.

#### Gaps

Significantly lower percentages of gaps for inhalation were observed in the intervention group with puff inhalers and dry powder capsules. Maximal gap length was significantly shorter for both, puff and dry powder capsules (Table 4). Sixteen participants assigned to the intervention with puff inhalers (16.4%) and four with dry powder capsules (4.1%) had no gaps during the whole study period. In the control group 14 participants with puff inhalers (12.8%) and two participants with dry powder capsules (1.8%) were identified without gaps.

**Table 4:** Mean percentage of gaps and maximal gap length

Variable	Intervention	Control	*P* value
Mean (SD) gaps for puff inhalers [%]	3.2 (4.7)^a^	11.7 (18.6)^c^	.008
Mean (SD) gaps for inhalation with dry powder capsules [%]	4.6 (4.4)^b^	9.8 (8.9)^d^	.009
Mean (SD) maximal gap length for puff inhalers [days]	1.6 (2.0)^a^	11.6 (25.6)^c^	.025
Mean (SD) maximal gap length for dry powder capsules [days]	2.6 (2.7)^b^	5.9 (5.2)^d^	.002

^a^*n* = 57; ^b^*n* = 41; ^c^*n* = 60; ^d^*n* = 49

#### Nonparametric test for time and group effects on adherence

The results of the nonparametric analysis of longitudinal data in factorial experiments are shown as online supplementary material (Additional file 3). Significant group effects were found for taking adherence with puff inhalers and dry powder capsules, and for timing adherence.

The group effect of the intervention group for taking and timing adherence is illustrated in Additional file 3. The larger effect in the intervention group is maintained throughout the whole study period and it is more clearly distinguishable for the taking adherence with puff inhalers (A) compared to the taking adherence with dry powder capsules (B). Similar results were observed for the timing adherence (C-D). A larger effect can be observed for the intervention group with a major difference for timing adherence with puff inhalers (C).

### Health-related QoL

Significant differences between the intervention and control groups were found at baseline regarding SGRQ total score and the subscale activity (Table 5). After 6 months, no significant differences in QoL were found between the two groups.

**Table 5:** Changes in SGRQ scores after 6 months

Variable	Symptoms	Activity	Impact	Total Score
Intervention				
Baseline (Mean (SD))	45.7 (21.5)	45.2 (19.3)	21.8 (14.6)	32.5 (14.7)
6-month change (95% CI)	−0.59 (4.3 to −5.5)	0.2 (3.9 to −3.5)	1.3 (4.7 to −1.9)	0.7 (3.7 to −2.4)
Control				
Baseline (Mean (SD))	48.7 (25.6)	52.4 (23.7)	29.3 (20.7)	39.6 (20.3)
6-month change (95% CI)	−2.9 (2.8 to −8.8)	0.1 (3.8 to −3.5)	− 2.0 (1.2 to −5.2)	− 1.5 (1.5 to −4.5)
*P* value				
Baseline	.44	.03	.05	.02
6 months change	.53	.70	.29	.77

*SD* Standard, *CI* Confidence interval

## Discussion

In this randomized controlled trial of an intervention promoting adherence to inhaled therapy a significant improvement in taking and timing adherence of inhaled medication could be demonstrated. A trend towards a decreased frequency of exacerbations was observed among participants in the intervention group.

No effect on time to next exacerbation or on the hazard of experiencing at least one exacerbation during the follow-up period was observed between the two study groups.

### Exacerbations during study period

The non-significant difference for the time to next exacerbation between the study groups could be explained by the follow-up period being too short or the participant number too low compared with other studies [30, 31]. Clinical studies also tend to include highly motivated participants causing a selection bias that influences health-outcomes in this type of study [32]. The current finding that participants in the intervention group experienced a trend towards a decreased frequency of exacerbations is supported by the fact that adherence in this group was significantly higher compared to the control group. This could also be shown in other studies, where high adherence, as in our study, was found to be associated with reduced exacerbation rates in asthma [10, 15, 16, 33] and COPD patients [17]. Furthermore, it could also be shown that patients with infrequent inhaler use had an increased rate of healthcare use. This, indicates that there is a clear association between adherence behaviours and clinical outcomes [34].

### Objective adherence

Adherence to inhaled medication has been investigated in a variety of clinical trials. However, the many of the studies conducted with asthma and COPD patients used prescription refill adherence or self-report measurements to assess the inhaled medication adherence [35]. The most frequently used adherence measure methods in the last 10 years were self-report measurements (38%), prescription refill data (33%) and electronic monitoring (19%) [35].

In comparison to previous studies applying subjective adherence measurements [36] or medication refill adherence [37], this study adds objectively acquired adherence data, which provide a more accurate view of the patient’s everyday adherence situation as well as of the effect of the applied intervention.

Adequate adherence to chronic therapies is of major importance for achieving therapeutic success. A Cochrane systematic review which analysed randomized controlled trials to improve adherence to pharmacological regimens showed that less than 50% of the interventions reached a significant improvement of adherence and only 30% demonstrated improved clinical outcomes [38]. For a successful intervention, the review suggested that frequent interaction with the patients focusing on adherence should be guaranteed. The results of this study confirm and further support this suggestion. Frequent interactions in form of patient-tailored, regular support calls whenever patients became non-compliant appear to represent a significant advantage compared to studies with limited and predefined follow-up appointments during the study period and can also explain the successful and significant improvement on the adherence achieved with this intervention. These findings are supported by a randomised clinical trial conducted by Sulaiman et al., which showed that repeated feedback as an intervention can significantly improve inhaler adherence [39].

Participants assigned to the intervention group had significantly better adherence. This shows that reminders can support patients to avoid forgetting to take their prescribed inhaled medication. These results are also confirmed by a systematic review which investigated the effect of electronic medication packaging devices on adherence indicating an increase of up to 34% [40]. Several other randomized controlled trials using electronic monitoring devices and reminder functions to study adherence to inhaled medication have likewise shown a significant improvement in adherence of different inhaled medication classes [41, 42]. Further, The improvement of medication adherence after a pharmacist intervention has already been confirmed in patients suffering from COPD [43] and asthma [44].

Furthermore, participants who inhaled with dry powder capsules had a higher percentage of days within the defined target range. This can be explained by the fact that medication available for dry powder capsules has a once-daily regimen and is thus easier to follow. This result is in line with other clinical trials which confirmed that taking and timing adherence were higher for once-daily regimens compared to two times or three times daily regimens [45]. The higher adherence rate obtained with electronic punch cards containing dry powder capsules might be explained by their simultaneous function as a visual reminder [46].

Overall, taking and timing adherence were relatively high for puff inhalers and dry powder capsules, within both study groups. A positive selection bias might be present which can influence the adherence. This could explain why participating patients already had a relatively high level of adherence and why it is therefore challenging to identify a positive effect on patients` health-outcomes [47, 48].

### Health-related QoL

The intervention showed no effect on QoL. The follow-up period may have been too short to detect a clinical significance. Moreover, the mean SGRQ total score for all participants was very low, generally indicating a good health status of the participants. Therefore, the possibility of reaching a clinically significant improvement is reduced. Similarly, other studies have observed comparable results when analysing health-related QoL after an intervention [43, 49]. However, studies that detected a significant improvement in QoL included a larger number of participants and all of them had a relatively poor QoL at baseline [50, 51]. The similar outcomes regarding QoL in both study groups can also be explained by a parallel effect of the study. Due to the fact that all participants received a general training course, experienced regular follow-up visits and had monitoring devices, it is likely that the control group also experienced a positive effect. Furthermore, wrong inhalation technique was corrected in all participants on ethical grounds, which is also likely to have influenced the results.

### Strengths and limitations

To our knowledge, our study is the first to investigate a population affected by asthma and/or COPD, which are different diseases however, certain important aspects in common. The aim to improve adherence to inhaled medication in order to achieve better outcomes, constitutes a common approach for these two diseases. Moreover, in previous similar studies of adherence and outcome, only the adherence to a preselected medication class was investigated. The current study, however, imposed no restrictions regarding medication class. This reflects a real-life situation for the participants, since they do not experience any drug changes due to the study. Second, there was real-time monitoring for the puff inhalers, which allowed a direct intervention without any delays. We acknowledge some limitations. First, adherence monitoring was unblinded. All participants were aware of the fact that adherence was measured throughout the whole study using the electronic devices. This could have caused a “Hawthorne effect” [52], which can elicit a bias in the data. Second, attention should be paid to the fact that two different lung diseases (asthma and COPD) were observed and analysed bearing the potential for unequal responses to medication, which may have affected the frequency of exacerbations in the study population. Third, the two study groups differ significantly concerning some baseline parameters that could have influenced the study results. In order to account for this imbalance, the analysis was adjusted for these parameters. Fourth, the relatively short follow-up period as well as the sample size might explain the partly non-significant study results.

Taken together, the results of this study demonstrate that the intervention is feasible in a study setting and associated with a significant improvement of the adherence to inhaled medication in patients with chronic lung diseases. Its implementation in developed countries should be considered because nowadays nearly everyone owns a smartphone where the app and consequently the reminders could easily be installed and used. However, the use of the adherence-measurement system and the described intervention currently add additional costs to standard care. Furthermore, clinicians will need to have sufficient time to review the adherence data and to provide appropriate feedback to the patients. Considering the high initial costs and the longer consultation times compared to the nowadays-established clinical visits, one should take into account that probably initial financial outlay and invested time for the patient care, a long-term saving can be achieved as well as an offsetting of the effort by improved disease control and consequently economic benefit. This point supports the findings of a systematic review published by R. Dekhuijzen et al., which summarised the results of different clinical trials investigating clinical and economic impact of adherence in asthma and COPD patients [8]. Therefore, further work is needed to confirm the value of this intervention in routine clinical practice.

## Conclusions

This study demonstrates that regular, automatic and personal reminders have a beneficial effect on taking and timing adherence of inhaled medication in asthma and COPD patients. This in turn, resulted in a trend towards a decreased frequency of exacerbation in participants assigned to the intervention group. A positive effect on time to first exacerbation as well as on QoL could not be detected. Further studies conducted in larger study populations and over extended follow-up periods are needed to verify our findings.

## Supplementary information

**Additional file 1.** Visualization graph for SmartinhalerTM and Polymedication Electronic Monitoring System.

**Additional file 2.** Results of adjusted Cox regression and Poisson regression.

**Additional file 3.** Nonparametric Test for Time and Group Effects on Adherence.

## Data Availability

The used raw data during this study are available from the corresponding author on reasonable request.

## Abbreviations

BMI: Body Mass Index
CI: Confidence Interval
COPD: Chronic Obstructive Lung Disease
FEV1: Forced Expiratory Volume in one Second
FVC: Forced Vital Capacity
GP: General Practitioner
ICS: Inhaled Corticosteroid
LABA: Long Acting Beta2-Agonist
LAMA: Long Acting Muscarinic Antagonist
POEMS: Polymedication Electronic Monitoring System
PY: Pack Years
QoL: Quality of Life
RR: Rate Ratio
SABA: Short Acting Beta2-Agonist
SAMA: Short Acting Muscarinic Antagonist
SD: Standard Deviation
SGRQ: St. George Respiratory Questionnaire
WHO: World Health Organisation
