# Supplementary material for: Does a tailored intervention to promote adherence in patients with chronic lung disease affect exacerbations? A randomized controlled trial
Source: Respir Res. 2019 Dec 3;20:273. doi: 10.1186/s12931-019-1219-3 (PMC6892023; doi:10.1186/s12931-019-1219-3)
Supplement: Supplementary file 1 — Additional file 1. Visualization graph for SmartinhalerTM and Polymedication Electronic Monitoring System. [file 12931_2019_1219_MOESM1_ESM.pdf]

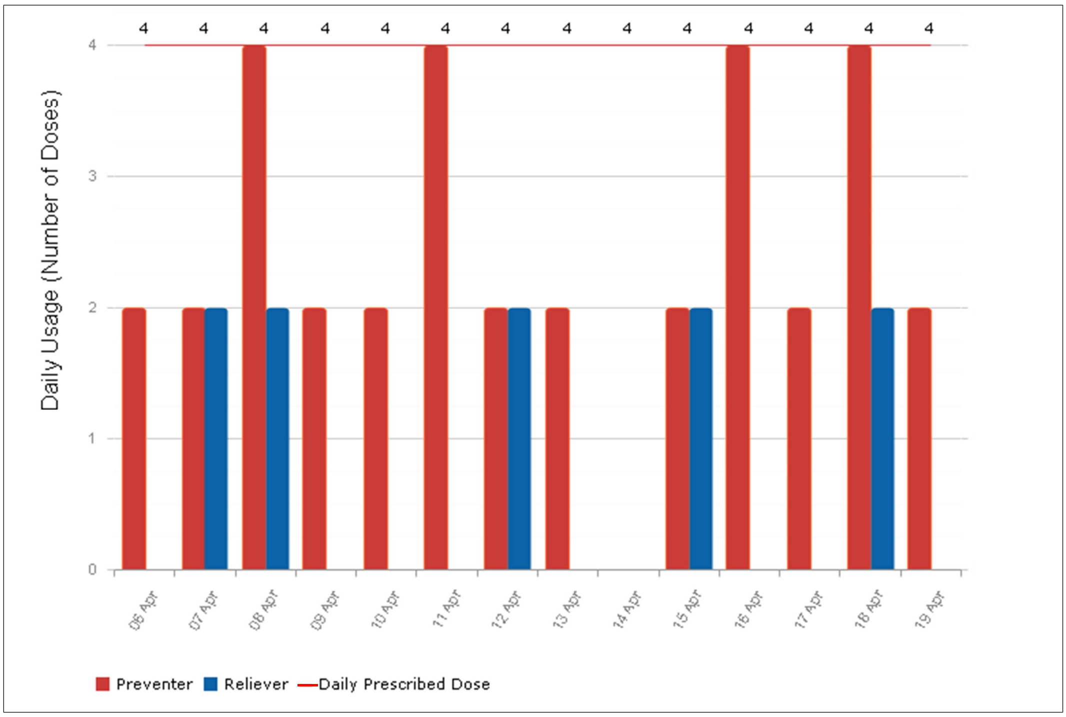

Output graph of a SmartInhaler™ adherence recoding. The example shows a typical tracing derived from a patient taking preventer medication (4x/d, 2-0-2) and reliever medication over the period of two weeks. On April 14th, a day without inhalation is detected. Red bars: preventer medication; blue bars: reliever medication.

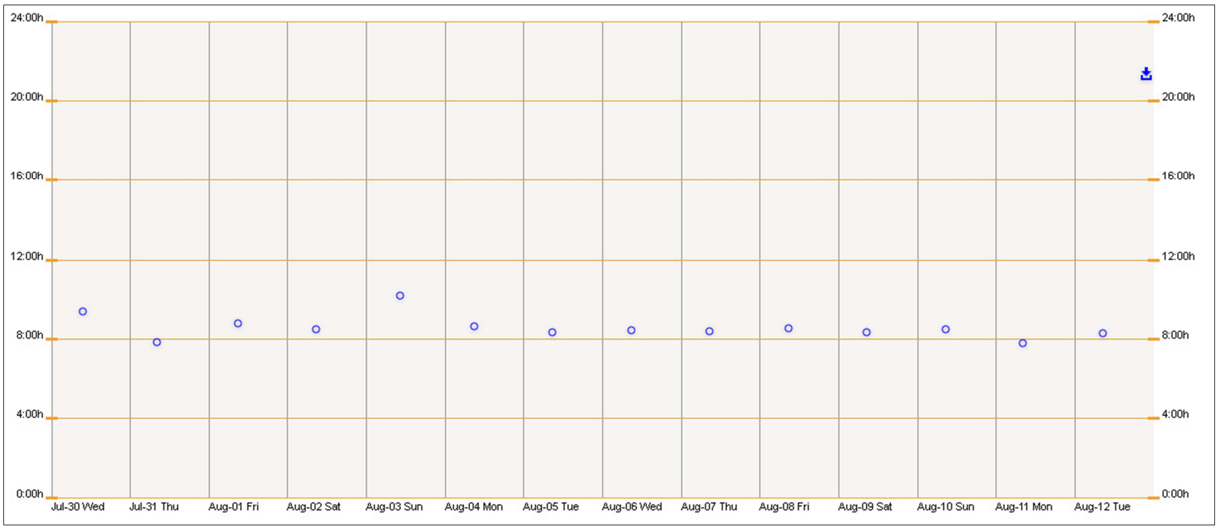

Output graph of a Polymedication Electronic Monitoring System (POEMS) adherence recording of a patient taking a once-daily medication regimen over the period of two weeks.
