# Supplementary material for: Does a tailored intervention to promote adherence in patients with chronic lung disease affect exacerbations? A randomized controlled trial
Source: Respir Res. 2019 Dec 3;20:273. doi: 10.1186/s12931-019-1219-3 (PMC6892023; doi:10.1186/s12931-019-1219-3)
Supplement: Supplementary file 2 — Additional file 2. Results of adjusted Cox regression and Poisson regression. [file 12931_2019_1219_MOESM2_ESM.pdf]

Baseline characteristic with the corresponding p-values

Characteristics	Intervention (n=75)	Control (n=74)	Chi squared/ t-test	Df	p-value
**Age**	64.7±12.4	69.0±8.8	-2.4562	133.11	**0.01533**
**Men**	46 (61)	51 (69)	0.639	1	0.4241
**Civil status**					
Unmarried	7 (9)	10 (14)	1.5127	2	0.4694
Married	46 (61)	48 (65)			
Divorced/widowed	22 (29)	16 (22)			
**Highest level of education at school [n]**					
Primary school	10 (13)	11 (15)	9.0191	4	0.06062
Apprenticeship	38 (50)	52 (70)			
Higher professional education	14 (19)	6 (8)			
University-entrance Diploma/Commercial college	2 (3)	1 (1)			
University / Collage of higher education	11 (15)	4 (6)			
**Employment status [n]**					
Active worker	23 (31)	15 (20)	3.9249	2	0.1405
Pensioner	47 (63)	57 (77)			
Never active	5 (7)	2(3)			
**Diagnosed lung disease**					
Asthma	30 (40)	16 (22)	6.4493	2	**0.03977**
COPD	32 (43)	45 (61)			
Asthma-COPD- overlap	13 (17)	13 (17)			
**Smoking status**					
Current smoker	16 (21.3)	12 (16.2)	2.0497	2	0.3589
Non-smokers	19 (25.3)	14 (18.9)			
Ex-smokers	40 (53.3)	48 (64.9)			
**Pack-years**	28.6±32.8	41.2±34.3	-2.2888	146.52	0.0235
**GOLD stage**					
1 (FEV_1>80% predicted), mild	2 (4)[a]	6 (10)[b]	1.6676	3	0.6442

2 (FEV_1 50-80% predicted), moderate	20 (45)[a]	24 (42)[b]			
3 (FEV_1 30-50% predicted), severe	19 (42)[a]	21 (36)[b]			
4 (FEV_1 <30% predicted), very severe	4 (9)[a]	7 (12)[b]			
**Co-existing illnesses [n]**					
Diseases of the cardiovascular system	44 (59)	46 (62)	0.0722	1	0.7882
Endocrine, nutritional and metabolic diseases	18 (24)	19 (26)	0.0022173	1	0.9624
Diseases of the gastrointestinal system	10 (13)	8 (11)	0.048846	1	0.8251
Diseases of the musculoskeletal system and connective tissue	16 (21)	16 (22)	0	1	1
**Medication [n]**					
LABA/LAMA combinations	10 (13)	9 (12)	0	1	1
LABA/ICS combinations	52 (69)	53 (72)	0.016	1	0.8993
LAMA	26 (35)	41 (55)	5.6628	1	0.01733
LABA	14 (19)	6 (8)	2.7225	1	0.09894
ICS	10 (13)	5 (7)	1.1272	1	0.2884
SABA	32 (43)	34 (46)	0.0566	1	0.8119
SABA/SAMA combinations	2 (2.7)	2 (2.7)	0	1	1
**Number of inhaled medication at baseline**					
1	22 (29.3)	23 (31.1)	2.5578	3	0.4649
2	36 (48.0)	27 (36.5)			
3	16 (21.3)	23 (31.1)			
4	1 (1.3)	1 (1.4)			

LAMA prescriptions and lung disease are strongly correlated. Therefore, we did not put the two variables LAMA and Lung Disease into the same multivariable model.

	Lama	
Lung disease	No [n]	Yes [n]
Asthma /COPD Overlap	16	10
Asthma Bronchiale	41	5
COPD	25	52

Pearson's Chi-squared test

X-squared = 37.9, df = 2, p-value = 5.89e-09

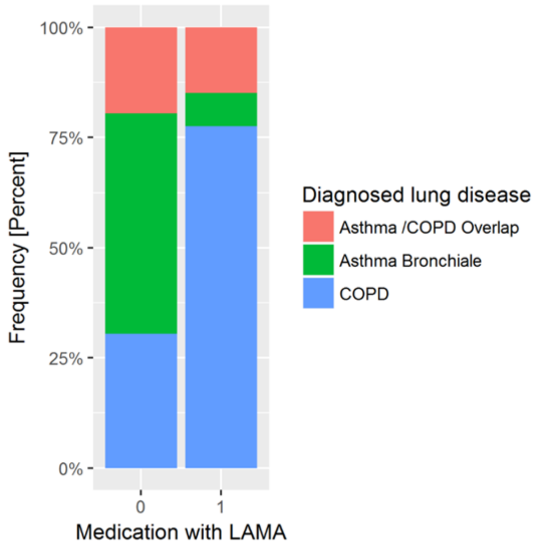

Further, age and LAMA are also significantly correlated, which is why we didn’t put these two variables in the same model.

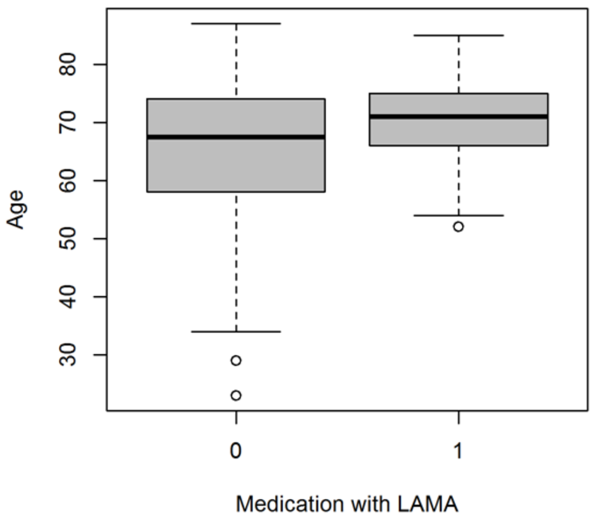

Welch Two Sample t-test

t = -2.9367, df = 135.99, p-value = 0.003897
alternative hypothesis: true difference in means is not equal to 0
95 percent confidence interval:
 -8.254889 -1.611146
sample estimates:
mean in group 0 mean in group 1
       64.63415   69.56716

In addition, packyears and LAMA are also correlated:.

```
        Wilcoxon rank sum test with continuity correction

data:  total$Pack_Years_Baseline by total$LAMA
W = 1789.5, p-value = 0.0002402
alternative hypothesis: true location shift is not equal to 0
```

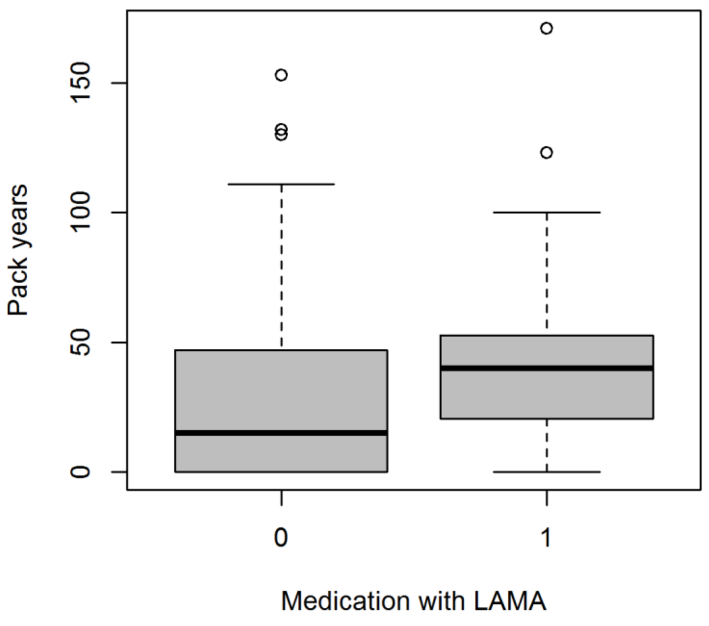

Results of the univariable Cox regression for time to next exacerbation.

	HR	95 % CI		Pr(>|z|)	Proportional Hazards assumption p-value	Overall-p-Value (using Likelihood ratio test comparing null model with model including categorical variable)
Age per 10 years	1.53	1.06	2.207	0.024	0.003	
Lung disease: Asthma /COPD Overlap vs. COPD	1.56	0.73	3.314	0.248	0.611	0.052
Lung disease: Asthma vs. COPD	0.47	0.19	1.164	0.103	0.049	0.052
Pack years per 10 years	1.11	1.02	1.201	0.015	0.723	
LAMA	2.487	1.266	4.887	0.008	0.584	
Cardiovascular.Comorbidity	1.166	0.593	2.289	0.656	0.054	
Endocrinologie.Comorbidity	0.957	0.452	2.028	0.909	0.436	
Gastrointestinal.Comorbidity	1.512	0.631	3.624	0.354	0.751	
muscles.comorbidity	0.672	0.280	1.611	0.373	0.790	

Multivariabel Cox regression results for group on time to next exacerbation.

		HR	95 % CI		Pr(>|z|)	Proportional Hazards assumption p-value
Model 1	Group: Control vs. Intervention	0.658	0.209	2.071	0.474	0.412
	Pack years	1.014	0.998	1.030	0.096	0.906
Model 2:	Group: Control vs. Intervention	0.862	0.441	1.684	0.664	0.142
	LAMA	2.397	1.195	4.805	0.014	0.381

The multivariabel model 1 was adjusted for age (strata), pack years, lung disease (strata).

Model 2 included group and LAMA since age, pack years and lung disease were associated with LAMA and multicollinearity would arise when including both variables in the same model. A strata of a variable was entered into the model if the variable did not meet the proportional hazards assumption.

Univariable Poisson regression.

	Incident rate ratios	95 % CI		Pr(>|z|)	Overall-p-Value (using Likelihood ratio test comparing null model with model including categorical variable)	Goodness of fit test for the overall model
Group: intervention vs. control	0.571	0.33	0.96	0.037		0.039
Age per 10 years	1.261	0.98	1.66	0.086		0.033
Gender: female vs. male	1.080	0.63	1.81	0.774		0.023
Pack Years per 10 years	1.060	0.989	1.128	0.082	0.032	1.060
Lung disease: Asthma /COPD Overlap vs. COPD	1.974	1.10	3.45	0.018	0.003	0.073
Lung disease: Asthma vs. COPD	0.558	0.26	1.10	0.110		0.073
LAMA	2.856	1.672	5.082	<0.001		0.115

Multivariable model of the association between incident exacerbations with study group adjusting for lung disease.

		Incident rate ratios	95 % CI		Pr(>|z|)	Overall-p-Value (using Likelihood ratio test comparing models including and excluding categorical variable)	Goodness of fit test for the overall model
Model 1	Group: Intervention vs. Intervention	0.610	0.353	1.031	0.070		0.091
	Lung disease: Asthma /COPD Overlap vs. COPD	2.055	1.149	3.599	0.013	0.005	0.091
	Lung disease: Asthma vs. COPD	0.627	0.288	1.253	0.208	0.005	0.091
Model 2	Group: Intervention vs. Intervention	0.696	0.401	1.177	0.184		0.123
	LAMA	2.649	1.534	4.760	0.001		0.123

The full model included either Lung disease (model 1) or LAMA (model 2) since both variables were significantly associated and multicollinearity would arise when including both variables in the same model.

In de main manuscript we have reported the results of model 1, since LAMA correlates strongly with age and packyears and these two variables are considerate in model 1.
