# Supplementary material for: Does a tailored intervention to promote adherence in patients with chronic lung disease affect exacerbations? A randomized controlled trial
Source: Respir Res. 2019 Dec 3;20:273. doi: 10.1186/s12931-019-1219-3 (PMC6892023; doi:10.1186/s12931-019-1219-3)
Supplement: Supplementary file 3 — Additional file 3. Nonparametric Test for Time and Group Effects on Adherence. [file 12931_2019_1219_MOESM3_ESM.pdf]

Related group and time effects and group: time effect interaction for taking and timing adherence with puff inhalers and dry powder capsules

	ANOVA-type Statistic	df	*p* value	Statistic	df	*p* value
	Puff inhalers (N=117)			Dry powder capsules (N=90)		
**Taking adherence**						
Group	6.3	1.0	**0.01**	7.9	1.0	**0.005**
Time	1.2	39.5	0.20	1.0	24.3	0.40
Group:Time	0.8	39.5	0.80	1.1	24.3	0.40
**Timing compliance**						
Group	15.3	1.0	**<0.001**	7.6	1.0	**0.006**
Time	1.1	36.14	0.30	1.3	29.9	0.10
Group:Time	0.9	36.14	0.60	1.2	29.9	0.20

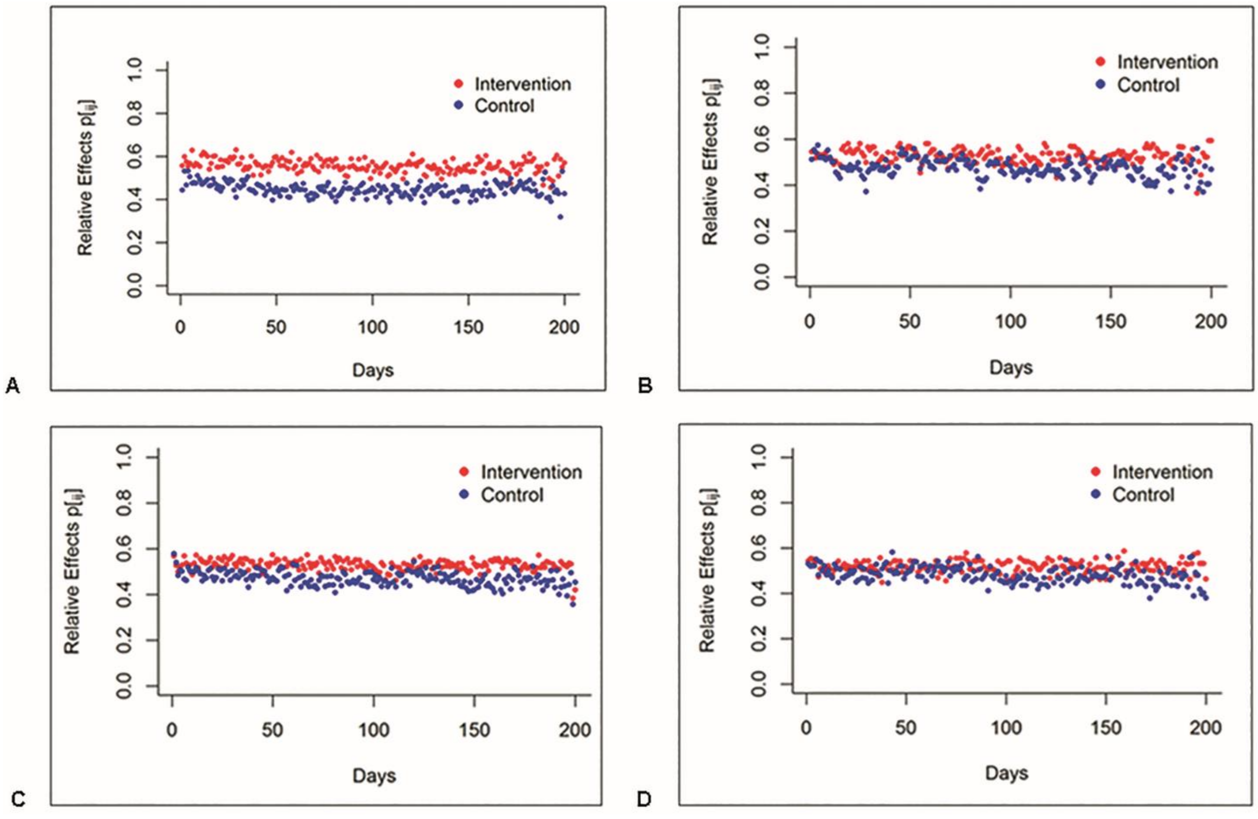

Group effect for the intervention and control group. Panel A: taking adherence with puff inhalers; Panel B: taking adherence with dry powder capsules. Panel C: timing adherence with puff inhalers; Panel D: timing adherence with dry powder capsules
